# Dysregulation of the immune microenvironment in essential thrombocythemia: the interplay of core genes and inflammation-related signaling pathways

**DOI:** 10.3389/fimmu.2026.1741533

**Published:** 2026-04-29

**Authors:** Yeqiong Li, Xiangguo Chen, Ying Guo, Rongrong Sha, Huicheng Hao, Jin Zhang, Honghong Song, Yuping Wei, Xiupeng Ye

**Affiliations:** 1 Department of Hematology, People’s Hospital of Ningxia Hui Autonomous Region, Yinchuan, Ningxia Hui Autonomous Region, China; 2 Department of Internal Medicine, Traditional Chinese Medicine Hospital of Xiji County, Guyuan, Ningxia Hui Autonomous Region, China; 3 Department of Radiotherapy, People’s Hospital of Ningxia Hui Autonomous Region, Ningxia Hui Autonomous Region, China; 4 Department of Radiology, Institute of Traditional Chinese Medicine of Ningxia Hui Autonomous Region, Yinchuan, Ningxia Hui Autonomous Region, China; 5 Department of Hematology, The Third Clinical College of Ningxia Medical University, Yinchuan, Ningxia Hui Autonomous Region, China

**Keywords:** diagnostic biomarkers, essential thrombocythemia, immune infiltration, machine learning, robust rank aggregation

## Abstract

**Background:**

Essential thrombocythemia (ET) is a myeloproliferative disorder characterized as excessive platelet production. Early and accurate diagnosis is critical to manage the disease and prevent progression to serious myeloid neoplasms like myelofibrosis or acute myeloid leukemia, though definitive diagnosis remains challenging.

**Materials and methods:**

We retrieved seven publicly available microarray datasets from GEO database. Data preprocessing included normalization and batch correction using limma and SVA packages. Differentially expressed genes (DEGs) were identified via robust rank aggregation (RRA). Protein-protein interaction networks, GO and KEGG analyses were performed. qRT-PCR was used to verify the expression of key genes in control subjects and patients with ET. Immune infiltration was analyzed with CIBERSORT, while logistic regression and LASSO models validated the diagnostic potential of the identified genes. Functional assays including shRNA knockdown, CCK-8, apoptosis detection, and Western blot were conducted in primary CD34+ cell-derived megakaryocytes from ET patients.

**Results:**

Eleven key DEGs were identified, and the logistic regression model achieved area under the curve of 0.846 and 0.863 for the training and test sets, respectively. Immune infiltration analysis revealed significant changes in B, T, and NK cells. The qRT-PCR analysis revealed *BP1*, *MMP8*, and *CEACAM8* up-regulated in ET patients. In particular, *LCN2* and *MMP8* were functionally validated: knockdown of either gene significantly reduced cell viability and promoted apoptosis in megakaryocytes. Further analysis showed that MMP8 knockdown was associated with reduced IL-17 signaling, while LCN2 knockdown led to inhibition of the NOD-like receptor pathway.

**Conclusion:**

This study identified an eleven-gene signature that links IL-17 and NOD-like receptor signaling to megakaryocyte dysfunction and immune dysregulation in ET. *In vitro* functional experiments suggested that LCN2 and MMP8 may contribute to megakaryocyte survival and inflammatory pathway activation. These findings provide a foundation for further investigation into the roles of LCN2 and MMP8 in ET pathogenesis and highlight them as candidates for future functional studies.

## 1 Introduction

Essential thrombocythemia (ET), a myeloproliferative disorder, is marked by massive production of platelets in the bone marrow (1). This disorder is commonly associated with genetic alterations such as JAK2, CALR, and MPL mutations, which lead to uncontrolled platelet production and increase the risk of life-threatening thrombotic events (2, 3). Therefore, accurate diagnosis, coupled with appropriate risk stratification and therapeutic interventions is essential for the management of ET. Clinical manifestations range from asymptomatic to the development of severe complications, emphasizing the importance of early diagnosis and treatment (4). Importantly, ET may progress to more serious myeloid neoplasms such as myelofibrosis or acute myeloid leukemia (5), so timely diagnosis is critical to mitigate these risks and optimize patient prognosis.

ET diagnosis requires excluding other myeloid neoplasms, which may be delayed without typical morphology or driver mutations (6). Molecular detection of gene mutations, particularly the *JAK2 V617F* mutation, presents difficulties in confirming the diagnosis to differentiate polycythemia vera from primary myelofibrosis (7). Compounding this complexity, ET exhibits significant molecular heterogeneity, with JAK2, CALR, and MPL mutations defining distinct clinical subtypes. JAK2 V617F is associated with a stronger pro-inflammatory profile and higher thrombosis risk, whereas CALR-mutated patients display higher platelet counts but lower inflammatory burden (8, 9). This heterogeneity underscores the need to investigate both mutation-specific and mutation-independent inflammatory mechanisms in ET. Recent advances in high-throughput sequencing technologies, combined with comprehensive bioinformatics tools, have improved diagnosis and patient stratification in a variety of diseases (10, 11). However, due to the small sample sizes of many microarray datasets, the batch effect poses a significant challenge for comprehensive analysis over multiple datasets. The robust rank aggregation (RRA) method method has been used in a wide variety of studies in this case to get the biomarkers that are stable (12). Machine learning algorithms have further extended diagnostic capabilities by identifying new genetic markers and constructing predictive models that can stratify patients according to their risk of developing complications (13). These computational tools, combined with high-throughput sequencing data, can identify complex mutation patterns that may be missed by traditional methods, significantly improving the diagnostic accuracy of ET (14).

The pathogenesis of ET thrombosis is multi-factorial, stemming from complicated interactions among blood cells, endothelial cells and the coagulation system (15). Elevated levels of erythrocytes, leukocytes, and platelets, along with quality anomalies, promote the thrombotic phenotype. In addition to these increased cell counts, *in vivo* evidence indicates neutrophil activation, demonstrated by elevated CD11b expression and increased circulating levels of elastase and myeloperoxidase, along with monocyte activation, as indicated by elevated CD25 expression (16). Emerging studies highlight the role of chronic inflammation in driving the hypercoagulable state in myeloproliferative neoplasms, suggesting that treatment addressing immune pathways may be a prospective strategy for mitigating ET-related thrombosis.

In this study, we performed an integrative analysis of seven publicly available ET microarray datasets using RRA and machine learning approaches. Our primary objectives were to identify consistently dysregulated genes across datasets as candidate diagnostic markers, characterize their association with immune infiltration patterns, and explore their potential functional roles through pathway enrichment analysis. By consolidating existing transcriptomic data, we aimed to confirm and extend current understanding of the inflammatory pathways—particularly IL-17 and NOD-like receptor signaling—that contribute to ET pathogenesis.

## 2 Materials and methods

### 2.1 Data acquisition

Microarray datasets for ET were retrieved from the publicly accessible Gene Expression Omnibus (GEO) database (https://www.ncbi.nlm.nih.gov/geo/). Datasets were selected based on the following criteria: (i) biology limited to “Homo sapiens”; (ii) datasets categorized as “expression analysis by array”; and (iii) studies that included control samples in the experimental design. Seven datasets met these criteria: GSE9827, GSE2006, GSE103237, GSE54644, GSE136335, GSE174060, and GSE61629. Detailed information for each dataset is provided in **Supplementary Table 1**.

### 2.2 Identification of core differentially expressed genes

The core DEGs were confirmed using two approaches. First, we utilized the limma package (17) to process the raw data from the seven datasets, which involved column normalization, log transformation, and variance analysis. This allowed us to generate ranked lists of up- and down-regulated DEGs in each dataset based on fold-change values (|logFC| > 1) and statistical significance (adjusted p-value < 0.05). The results from these datasets were then integrated using the R package RobustRankAggreg (18), which employs the RRA method to analyze the most-significant DEGs. Genes with |logFC| > 1 and an adjusted p-value < 0.05 were considered significant in the RRA analysis.

Second, to account for potential batch effects arising from differences in laboratory conditions, reagent batches, and personnel, we utilized the SVA package (19) and its ComBat function to normalize the gene expression data across datasets. To assess correction effectiveness, we generated boxplots and PCA plots before and after adjustment, expecting comparable expression distributions and elimination of batch-driven clustering post-correction. Following batch effect removal, DEGs were re-screened from the normalized merged matrix using the limma package with a stringent threshold (|logFC| > 1, adjusted P-value < 0.05) to ensure high confidence for downstream analysis.

### 2.3 Protein-protein interaction and gene enrichment analysis

GO and KEGG analysis of ET core genes was performed with the R package clusterProfiler (20). PPI maps were constructed on STRING (https://string-db.org/), and the results were presented using Cytoscape.

### 2.4 Sample collection and qRT-PCR assay

The blood samples (20 ET and 20 control) were collected from People’s Hospital of Ningxia Hui Autonomous Region, and the diagnostic criteria for the patient followed the WHO classification published in 2022 (21). All participants have signed the informed consent, and the protocol has received approve from Institutional Ethics Committee of People’s Hospital of Ningxia Hui Autonomous Region (2021-NZR-067). Total RNA was prepared using Trizol (Invitrogen) and concentration determined on a Nanodrop 2000 (ThermoFisher Scientific). HiScript II 1st Strand cDNA Synthesis Kit (Vazyme) were used for reverse transcription. SYBR Master Mix (Cwbio) was used to construct the reaction system, and PCR was carried out using the ABI7500 platform (ThermoFisher Scientific), with GAPDH as the internal reference, and the primers were listed in **Supplementary Table 2**.

### 2.5 Immune infiltration analysis and machine learning algorithms

To evaluate immune activity in ET, we used CIBERSORT (22) to quantify immune cell classes, corrplot R packag to visualize their correlation with core genes. For machine learning analysis, logistic regression was applied to model core genes for ET diagnosis, while LASSO was used to identify key immune cell subtypes.

### 2.6 Cell culture and transfection

Primary hematopoietic stem cells (HSCs) were separated from peripheral blood of ET patients meeting WHO criteria. Mononuclear cells were enriched by Ficoll-Paque PLUS (Cytiva) gradient centrifugation, and purified using magnetic-activated cell sorting (MACS) CD34 microbeads (Miltenyi Biotec) to obtain CD34+ cells. The purified CD34+ cells were cultured in serum-free medium (Yeasen) supplemented with recombinant human thrombopoietin (50 ng/mL), stem cell factor (20 ng/mL), IL-3 (10 ng/mL) and 1% penicillin-streptomycin (Sigma-Aldrich) to induce megakaryocyte differentiation, and the cells were maintained in a 5% CO_2_ environment at 37 °C for 14 d with medium changes every 3 d. CD41/CD61 flow cytometric analysis was used to verify the differentiation.

For knockdown experiments, *LCN2* and *MMP8*-specific shRNAs and scrambled controls were designed and cloned into lentiviral vectors. Lentiviruses were produced in 293T cells using standard packaging plasmids. Megakaryocytes were transduced with the respective lentiviruses and screened using puromycin (2 µg/mL) to produce stabilized cell lines.

### 2.7 Cell functional assays​

Cell Counting Kit-8 (CCK-8) Assay: Cell viability was analyzed using the CCK-8 kit (Dojindo). Cells were seeded in 96-well plates and exposed to cisplatin (CDDP; Selleckchem) for 24–72 hours. Added CCK-8 reagent to each well, and the 450 nm absorbance was measured.

Flow cytometry: Apoptosis was assessed using an Annexin V-FITC/PI double-staining kit (Invitrogen). Briefly, cells were harvested, washed, and then adjusted to 1×10^6^ cells/mL with 1× binding buffer. 100 μL cells was taken plus 5 μL Annexin V-FITC with 5 μL PI, protected from light for 15 min, and then replenished with 400 μL 1× binding buffer, and immediately put on a flow cytometer (BD Biosciences). Data processing was performed using FlowJo software.

### 2.8 Western blotting analysis

Cell lysis was performed in RIPA buffer (Beyotime), and the concentration was determined using the BCA kit (Pierce). SDS-PAGE was used to separate the samples and then the proteins were transferred to PVDF membranes (Millipore), blocked with 5% skimmed milk. The primary antibodies including IL-17RA, IL-17A, NOD1, NOD2, NF-κB, RIP2, and GAPDH (Abcam) were used to incubate the samples at 4 °C overnight. HRP-labeled secondary antibodies were added, and developed using the ECL system (Tanon), with GAPDH was used as an internal reference.

### 2.9 Statistical analysis

Correlations were statistically estimated by Spearman/Pearson analysis. Inter-group comparisons were performed with the Wilcoxon’s test, and significance was set at *P* < 0.05. All data analyses were implemented at R 4.0.3 platform.

## 3 Results

### 3.1 Identification of significantly DEGs by RRA

**Figure 1:**
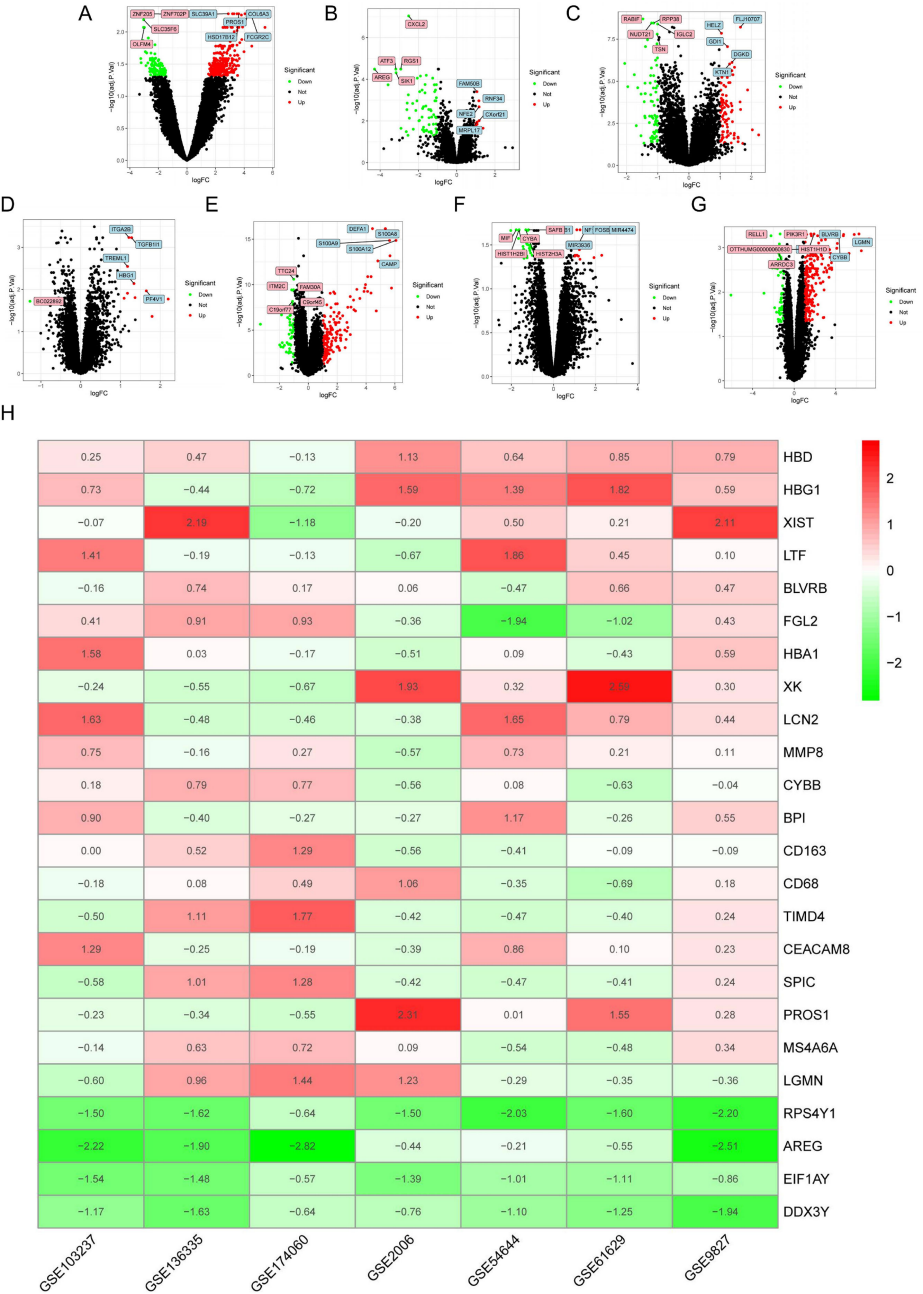
The screening of DEGs in ET patients and control samples. The top 5 most significantly DEGs were shown. These include **(A)** GSE2006, **(B)** GSE9827, **(C)** GSE54644, **(D)** GSE61629, **(E)** GSE103237, **(F)** GSE136335 and **(G)** GSE174060. **(H)** Heatmap of genes in RRA analysis for 7 datasets. Columns correspond to datasets and rows denote genes.

DEGs were analyzed for each dataset according to the steps in the methodology (**Figures 1A–G**), and 26 up-regulated and 4 down-regulated DEGs were subsequently identified with the RRA method (**Figure 1H****;****Supplementary Table 3**).

### 3.2 Analysis of overall data after batch correction

**Figure 2:**
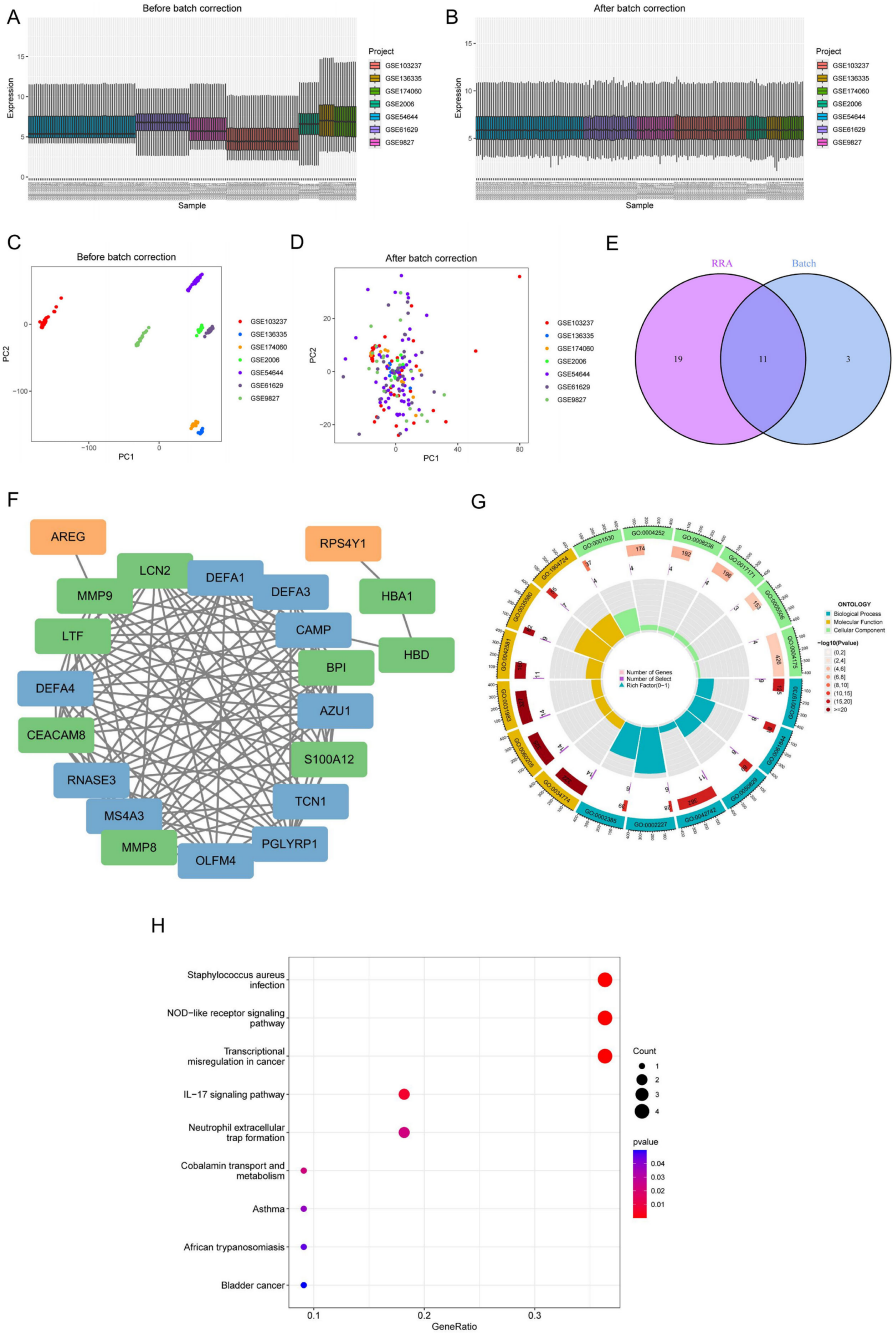
Analysis of enrichment of key genes and protein interaction networks. **(A, B)** Analysis of variance of data after correction for batch effects. **(C, D)** PCA cluster plots after correction for batch effects. **(E)** Intersection genes after RRA analysis and integrated data matrix analysis. **(F)** PPI network of DEGs, green represents up-regulation, orange represents down-regulation, and blue represents that they are reciprocal proteins. **(G)** GO enrichment analysis. **(H)** KEGG enrichment analysis.

After removing the batch effect, the seven datasets encompassed a total of 109 ET samples and 65 control samples. **Figures 2A, B** demonstrates the merged gene expression matrices presented in the box plots after correction and after correction. After normalization and adjustment for batch effects, there was no significant batch effect between the different datasets, as reflected in the results of the PCA analysis (**Figures 2C, D**). After differential analysis, a total of 14 DEGs were obtained. The intersection of these 14 DEGs with the 30 DEGs identified by RRA yielded 11 core genes (**Figure 2E**).

### 3.3 Analysis of PPI and functional enrichment

Protein-protein interaction networks of the core DEGs were constructed using STRING and visualized with Cytoscape (**Figure 2F**). These genes were included in GO analysis, and the pathway was significantly enriched in lipopolysaccharide binding, serine hydrolase activity, iron ion binding, and endopeptidase activity (**Figure 2G**). Further KEGG analysis showed that the differentially significant genes enriched at the NOD- like receptor, IL-17 and neutrophil extracellular trap formation pathway (**Figure 2H**).

### 3.4 Assessment of the diagnostic potential of core genes

**Figure 3:**
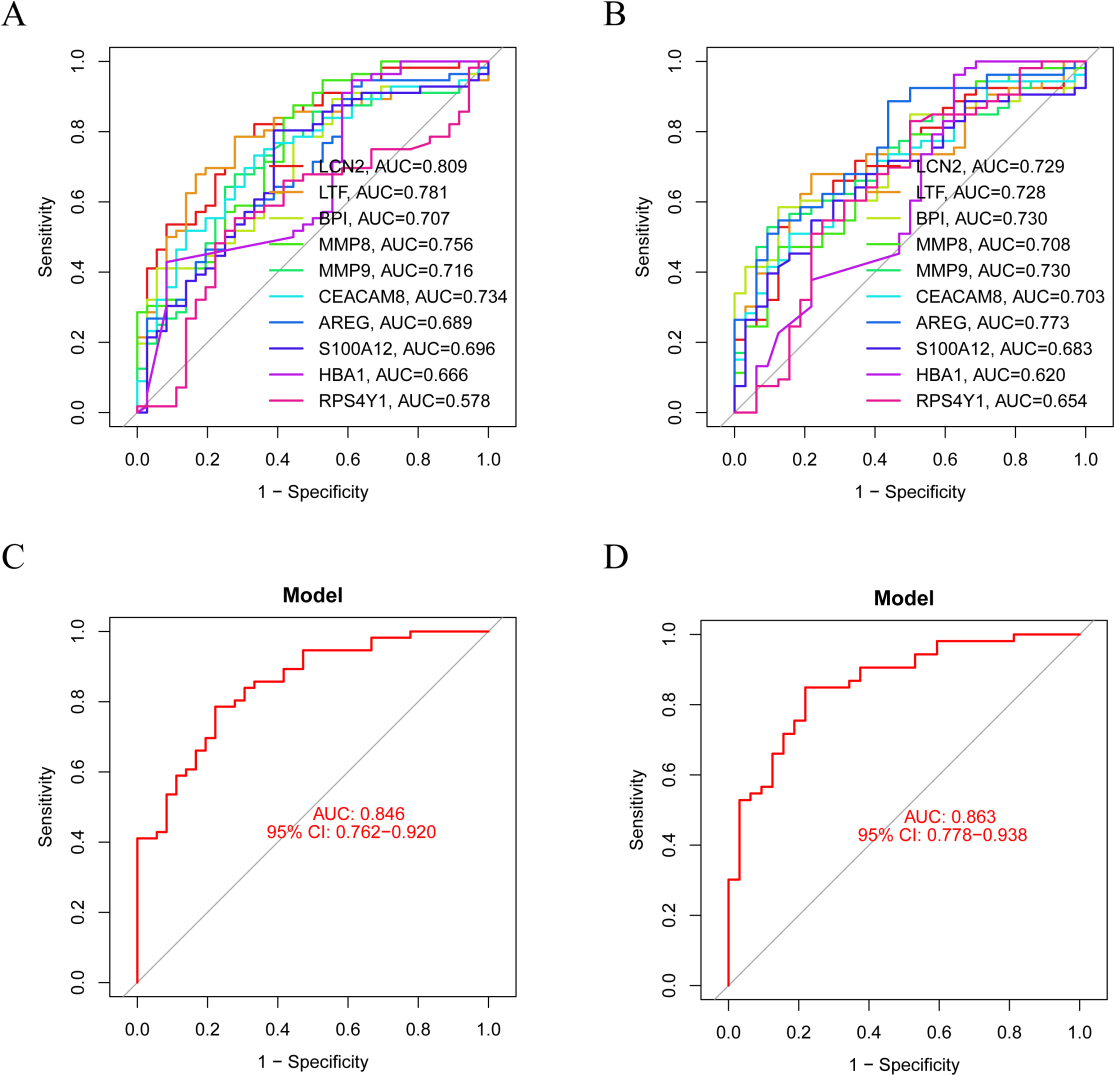
Validation of diagnostic performance. ROC curves for individual core genes of **(A)** training set and **(B)** test set. ROC curves for logistic regression models in **(C)** training set and **(D)** test set.

**Figure 4:**
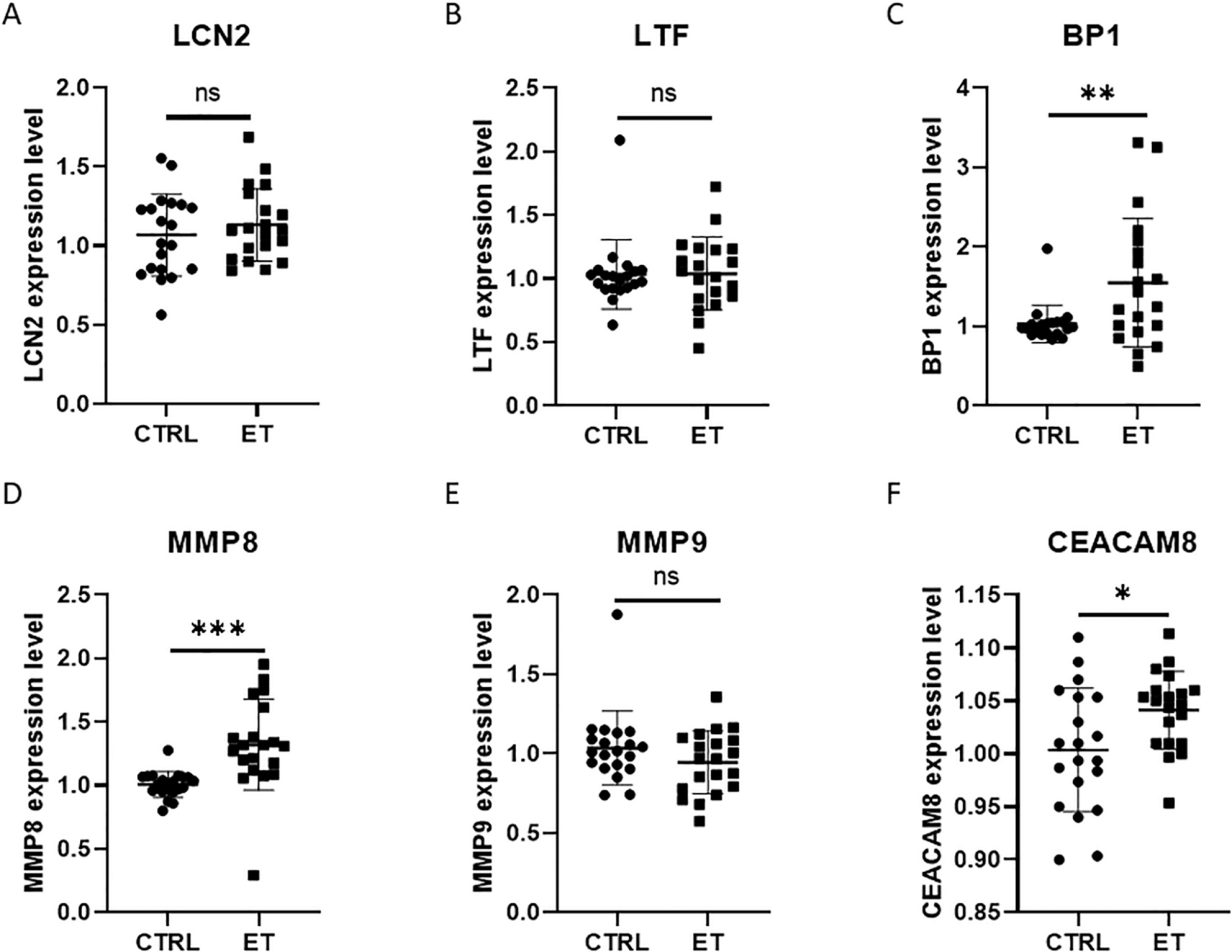
Analysis of qRT-PCR. mRNA level of *LCN2***(A)**, *LTF***(B)**, *BP1***(C)**, *MMP8***(D)**, *MMP9***(E)**, and *CEACAM8***(F)** in different groups. *P<0.05, **P<0.01, ***P<0.001; ns, not significant.

To determine the diagnostic efficacy of the identified gene signature for ET. We stochastically parted the matrix into training and test sets. The area under the curve (AUC) of *LCN2*, *LTF*, *BPI*, *MMP8*, *MMP9*, *CEACAM8* was greater than 0.7 in both the training and test sets, suggesting their possible potential as diagnostic markers (**Figures 3A, B**). Further, we integrated all core genes and executed logistic regression modeling, and found that the model constructed for all core genes had better diagnostic performance and generalizability, with AUC of 0.846 and 0.863 in the training and test sets (**Figures 3C, D**). CD34+ hematopoietic stem cells (HSCs) isolated from ET and control peripheral blood were differentiated into megakaryocytes, and qRT-PCR analysis confirmed significant differential expression of BPI, MMP8, and CEACAM8(P < 0.05; **Figures 4A–F**).

To assess whether the diagnostic potential of these core genes is mutation-dependent, we performed stratified analysis in GSE103237. Most core genes (LTF, BPI, MMP8, MMP9, CEACAM8, LCN2, S100A12, AREG, HBD) were significantly dysregulated in both JAK2- and CALR-mutated ET versus controls (P < 0.01), with no significant differences between subtypes (P > 0.05; **Supplementary Figure 1**). This suggests the gene signature reflects mutation-independent transcriptional features of ET, supporting its broad diagnostic applicability.

### 3.5 Immunoinfiltration analysis of ET

**Figure 5:**
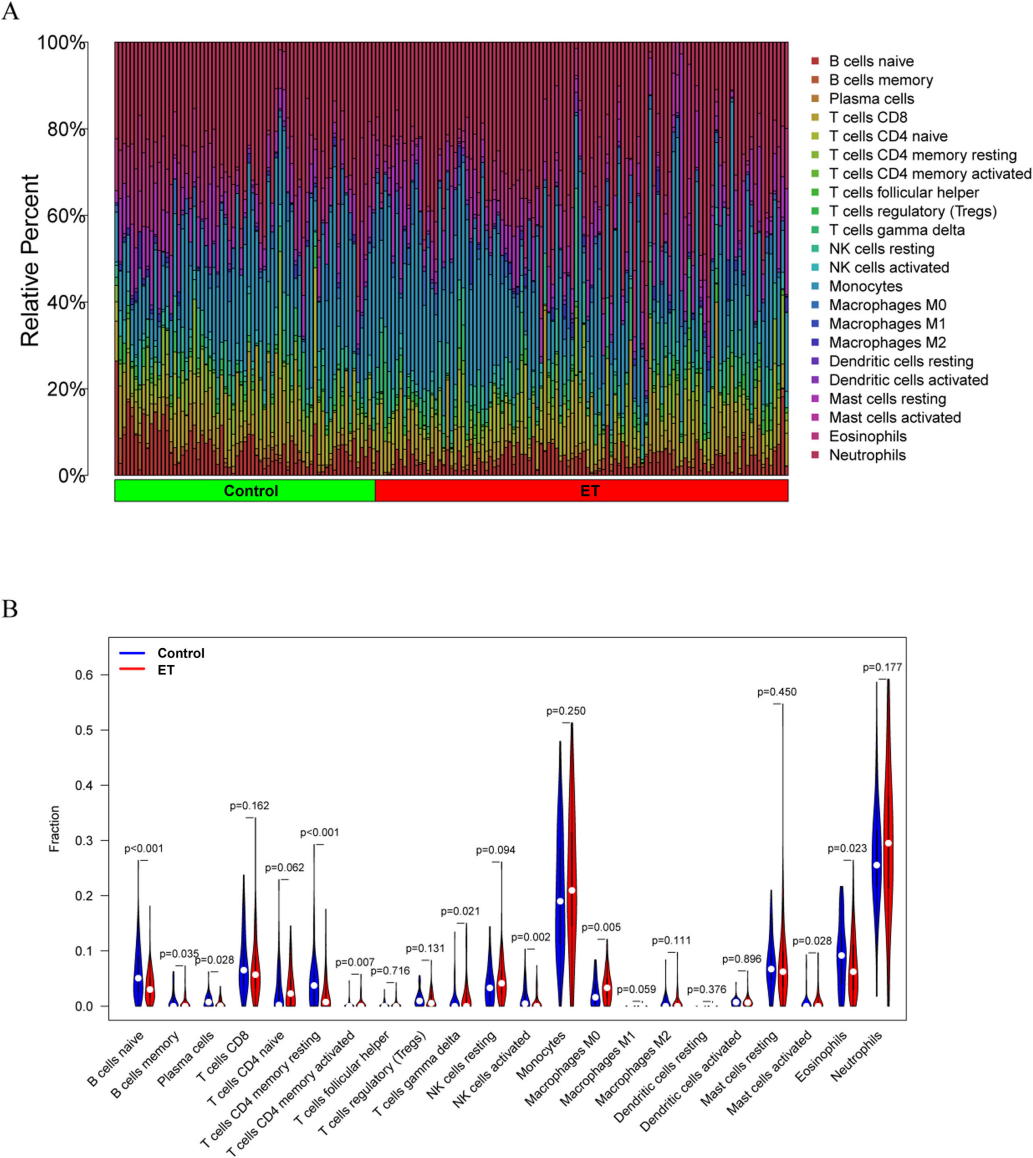
Assessment and visualization of immune infiltration in ET. **(A)** Calculation of 22 immune cell ratios. **(B)** Comparative analysis of differences in immune cells.

**Figure 6:**
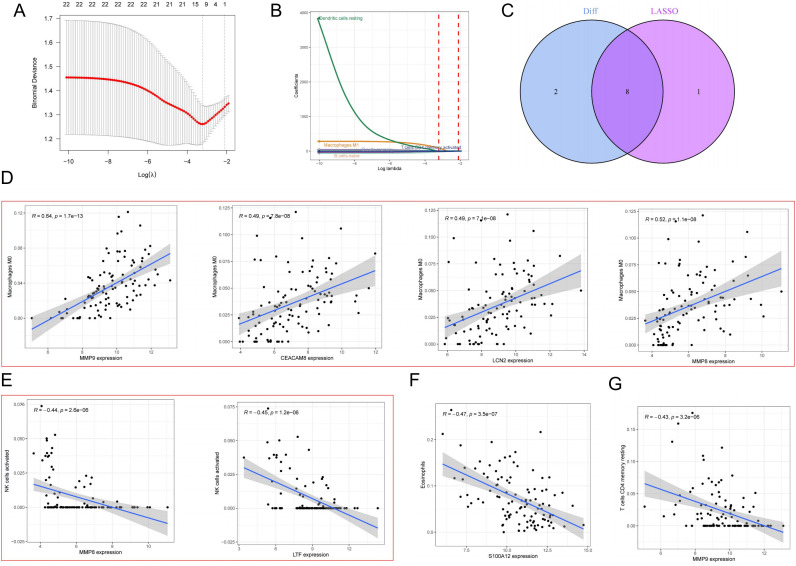
Correlation analysis of ET-related immune cell subtypes. **(A)** Least Absolute Shrinkage and Selection Operator (LASSO) logistic regression algorithm to screen the best immune cell features. **(B)** Screening of λ values. **(C)** Difference-in-difference analysis and VENN plots of lasso screening methods. **(D)** Correlation analysis of macrophage M0 with *MMP9*, *CEACAM8*, *LCN2*, and *MMP8*. **(E)** Correlation analysis of NK cell activation with *MMP8* and *LTF*. **(F)** Correlation analysis of eosinophis with *S100A12*. **(G)** Correlation analysis of T cells CD4 memory resting with *MMP9*.

Using CIBERSORT, we estimated the proportions of 22 immune cell subtypes in ET and control samples (**Figure 5A****;****Supplementary Table 4**). Compared to controls, ET samples exhibited lower estimated proportions of eosinophils, naive B cells, resting memory CD4 T cells, plasma cells, and activated NK cells, and higher estimated proportions of gamma delta T cells and activated memory CD4 T cells (**Figure 5B**). LASSO regression identified 9 ET-associated immune cell features (**Figures 6A, B**), with 8 overlapping the differentially abundant cells (**Figure 6C**). Correlation analyses showed that macrophage M0 estimates were positively correlated with MMP9, CEACAM8, LCN2, and MMP8 expression (**Figure 6D****;****Supplementary Table 5**). MMP8 and LTF were negatively correlated with activated NK cell estimates (**Figure 6E**), S100A12 with eosinophil estimates (**Figure 6F**), and MMP9 with resting memory CD4 T cell estimates (**Figure 6G**).

### 3.6 *In vitro* functional verification of the core genes LCN2 and MMP8

**Figure 7:**
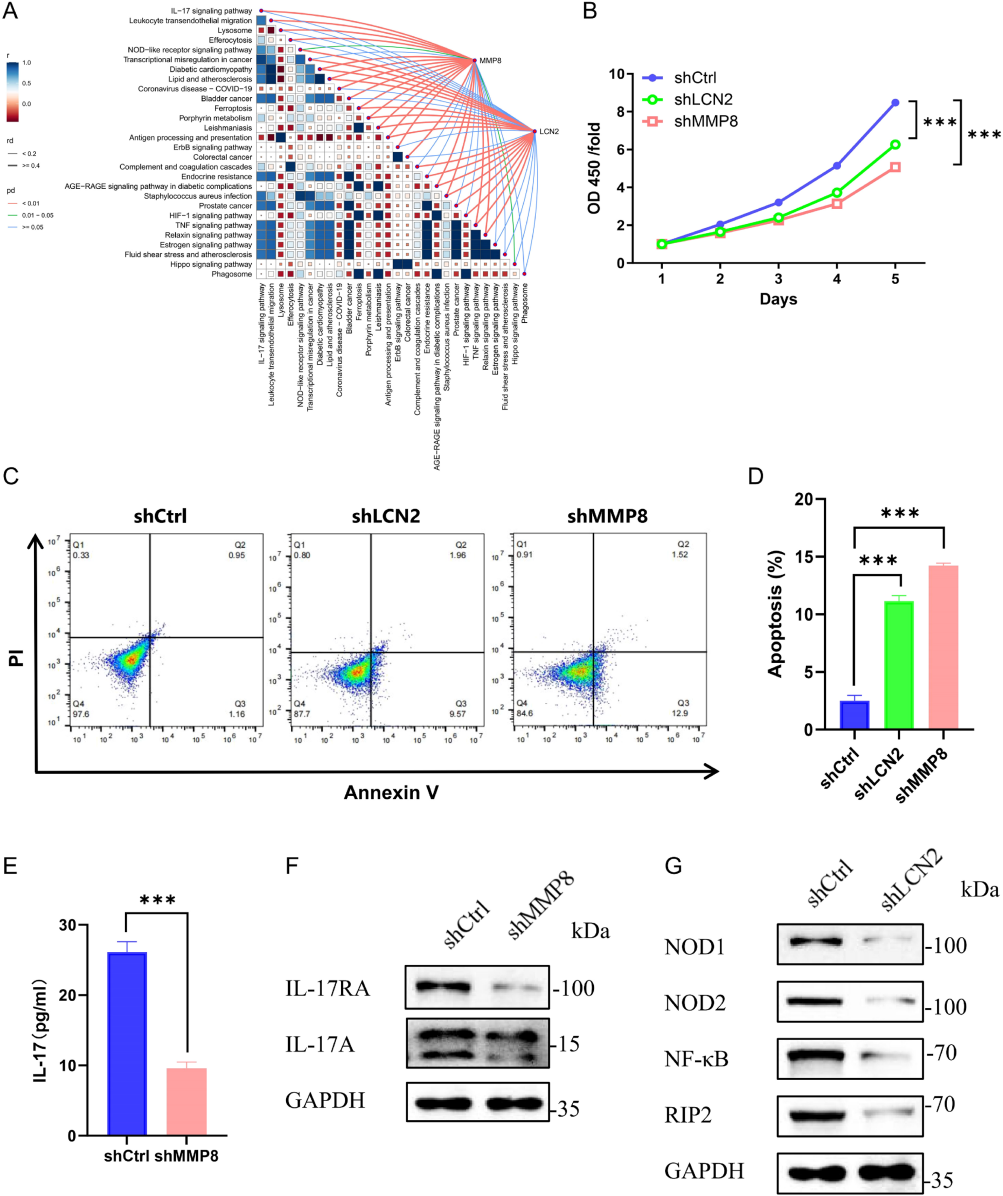
In vitro functional verification of the core genes LCN2 and MMP8. **(A)** Gene-pathway correlation analysis of LCN2 and MMP8. **(B)** Cell viability detection of CCK-8 assay. **(C)** Apoptosis was assessed by flow cytometry. **(D)** The statistics of the proportion of cell apoptosis. **(E)** ELISA was used to detect the concentration of IL-17. **(F)** Representative Western blot analysis of IL-17RA, IL-17A proteins in megakaryocytes cells transfected with shCtrl or shMMP8. **(G)** Representative Western blot analysis of NOD1, NOD2, NF-κB, and RIP2 proteins in megakaryocytes cells transfected with shCtrl or shLCN2. ***P<0.001.

Based on the above analysis, we selected LCN2 and MMP8 for further functional investigation *in vitro*. Gene-pathway correlation analysis revealed an association between MMP8 and the IL-17 signaling pathway, while LCN2 was significantly correlated with the NOD-like receptor signaling pathway (**Figure 7A**), consistent with prior KEGG enrichment results. We then isolated HSCs from patients with ET and differentiated them into megakaryocytes. These cells were infected with lentivirus expressing shRNA targeting *LCN2* or *MMP8*. Compared to the control group, knockdown of *LCN2* or *MMP8* significantly suppressed cell viability (**Figure 7B**). Moreover, loss of either gene markedly promoted apoptosis in megakaryocytes (**Figures 7C, D**). Further validation of the predicted pathways showed that MMP8 knockdown markedly reduced IL-17 secretion and its receptor expression (**Figures 7E, F**). Similarly, *LCN2* knockdown downregulated key proteins within the NOD-like receptor signaling pathway, including NOD1, NOD2, NF-κB, and RIP2 (**Figure 7G**). Taken together, these *in vitro* findings provide further evidence supporting the involvement of LCN2 and MMP8 in megakaryocyte survival and inflammatory pathway activation in ET, consistent with their established roles as mediators of neutrophil activation and tissue remodeling. Nevertheless, their precise functions in disease pathogenesis *in vivo* warrant further investigation.

## 4 Discussion

Complete blood counts, bone marrow biopsies, and cytogenetic analyses remain the cornerstone of the WHO classification system for ET diagnosis and are essential for confirming ET and excluding reactive causes (23). Nevertheless, these criteria do not fully capture the molecular heterogeneity of the disease. Consequently, there is a growing need for robust, disease-specific biomarkers that can refine risk stratification and illuminate the pathogenic mechanisms amenable to targeted therapy. In this study, a comprehensive bioinformatics analysis was performed using seven publicly available ET microarray datasets. By combining RRA with integrated matrix differential analysis, we identified 11 key genes in ET. Among these, LCN2, LTF, BP1, MMP8, MMP9, and CEACAM8 emerged as potential diagnostic markers. Logistic regression modeling based on the 11-gene signature demonstrated effective diagnostic performance. In addition, immune infiltration profiling was employed to examine the relationships between key genes and immune cell subsets in ET.

LCN2 plays a core role in iron transport and inflammation. Levels of this myeloid-derived factor are significantly elevated in ET plasma and contribute to excessive ROS production, leading to DNA double-strand breaks and apoptosis in CD34+ cells (24, 25). Excess LCN2 has been reported to trigger a cascade that promotes myelofibrotic progression (26). Its rapid stimulation in MSCs first upregulates RUNX2, followed by elevated TGF-β and BMP2, accelerating osteoclast expansion and remodeling the fibrotic bone marrow microenvironment (27, 28). LTF is another iron-binding protein engaged in immune response and antimicrobial defense. Our results showed a negative relationship with the activation of NK cells (R = -0.45, P = 1.2e-06). NK cells play a critical role in early defense and immune surveillance by rapidly destroying tumor or virus-infected cells and releasing IFN-γ and TNF-α, which influence innate and adaptive immune development (29, 30). Several studies have reported lower proportions of NK cells in untreated patients (excluding therapeutic bloodletting) compared to healthy donors (31), which may be associated with increased LTF expression in ET.

MMP8 and MMP9 belong to the matrix metalloproteinase family, which degrade extracellular matrix components and play key roles in inflammatory responses and tissue remodeling (32). Recent studies have reported increased MMP9 expression in ET plasma samples (33). Consistently, our analysis of both myeloid and whole blood data showed that MMP9 was overexpressed in ET and correlated with macrophages and T cells. Notably, IL-17 and NOD-like receptor signaling pathways were significantly enriched among the differentially expressed genes in our KEGG analysis, with MMP9 contributing to this enrichment. From a therapeutic perspective, it is noteworthy that Lcn2 and MMP8 knockout mice are viable with no overt hematopoietic defects under basal conditions (34, 35), suggesting that targeting these genes may have a favorable safety profile. Nevertheless, direct validation in human healthy donor cells remains necessary.

This study has the following limitations: first, the proportion of immune cells is inferred from transcriptome data using the CIBERSORT algorithm, which is a computational estimate rather than a direct quantitative finding, and lacks experimental confirmation such as flow cytometry. Thus, conclusions about changes in NK cells and T cell subsets should be viewed as hypothetical generative results. Second, the extent to which public data annotation is complete limits mutation stratification analyses. Only GSE103237 has JAK2 and CALR mutation information, and the CALR mutation negative group in this dataset has a limited sample size (n=7). At the same time, there is a lack of MPL mutation data and clinical outcome information such as thrombosis, fibrosis transformation, and survival rate. Third, in the functional validation experiment, due to a limitation in the source of primary CD34+cells, LCN2 and MMP8 knockout controls were not performed in parallel in healthy donor cells, and their effects on normal megakaryocytes remain unknown. In the end, the outcomes of this study should be interpreted as exploratory and speculative, providing preliminary clues for further research. In conclusion, we utilized RRA, integrated matrix analysis and machine learning to identify critical genes associated with ET. Our analysis suggests that immune infiltration plays a crucial role in ET. These findings validate and consolidate existing evidence on inflammatory pathways in ET, reinforcing the potential of LCN2 and MMP8 as candidates for further investigation. However, additional studies are needed to further validate these results and elucidate the precise mechanisms by which immune infiltration contributes to ET pathogenesis, building upon the consolidated evidence provided by this study.

## Data Availability

The original contributions presented in the study are included in the article/**Supplementary Material**. Further inquiries can be directed to the corresponding authors.

## References

[1] Alimam S Wilkins BS Harrison CN . How we diagnose and treat essential thrombocythaemia. Br J Haematol. (2015) 171:306–21. doi: 10.1111/bjh.13605, PMID: 26464262

[2] Godfrey AL Green AC Harrison CN . Essential thrombocythemia: challenges in clinical practice and future prospects. Blood. (2023) 141:1943–53. doi: 10.1182/blood.2022017625, PMID: 36379024

[3] Tefferi A Vannucchi AM Barbui T . Essential thrombocythemia: 2024 update on diagnosis, risk stratification, and management. Am J Hematol. (2024) 99:697–718. doi: 10.1002/ajh.27216, PMID: 38269572

[4] Tefferi A Barbui T . Polycythemia vera and essential thrombocythemia: 2017 update on diagnosis, risk-stratification, and management. Am J Hematol. (2017) 92:94–108. doi: 10.1002/ajh.24607, PMID: 27991718

[5] Barbui T Carobbio A . Prediction models for essential thrombocythemia from two longitudinal studies involving 2000 patients. Blood Cancer J. (2024) 14:17. doi: 10.1038/s41408-024-00987-y, PMID: 38253717 PMC10803320

[6] Leonard JP Martin P Roboz GJ . Practical implications of the 2016 revision of the world health organization classification of lymphoid and myeloid neoplasms and acute leukemia. J Clin Oncol. (2017) 35:2708–15. doi: 10.1200/JCO.2017.72.6745, PMID: 28654364

[7] Tefferi A . Primary myelofibrosis: 2021 update on diagnosis, risk-stratification and management. Am J Hematol. (2021) 96:145–62. doi: 10.1002/ajh.26050, PMID: 33197049

[8] Saki N Shirzad R Rahim F Saki Malehi A . Estimation of diagnosis and prognosis in ET by assessment of CALR and JAK2(V617F) mutations and laboratory findings: a meta-analysis. Clin Transl Oncol. (2017) 19:874–83. doi: 10.1007/s12094-017-1618-1, PMID: 28205126

[9] Tefferi A Vannucchi AM Barbui T . Essential thrombocythemia treatment algorithm 2018. Blood Cancer J. (2018) 8:2. doi: 10.1038/s41408-017-0041-8, PMID: 29321520 PMC5802626

[10] Sanchez-Carvajal JM Rodriguez-Gomez IM Ruedas-Torres I Zaldivar-Lopez S Larenas-Munoz F Bautista-Moreno R . Time series transcriptomic analysis of bronchoalveolar lavage cells from piglets infected with virulent or low-virulent porcine reproductive and respiratory syndrome virus 1. J Virol. (2022) 96:e0114021. doi: 10.1128/JVI.01140-21, PMID: 34851149 PMC8826917

[11] Gao M Kong W Huang Z Xie Z . Identification of key genes related to lung squamous cell carcinoma using bioinformatics analysis. Int J Mol Sci 21. (2020) 21:2994. doi: 10.3390/ijms21082994, PMID: 32340320 PMC7215920

[12] Ran Z Mu BR Zhu T Zhang Y Luo JX Yang X . Predicting biomarkers related to idiopathic pulmonary fibrosis: Robust ranking aggregation analysis and animal experiment verification. Int Immunopharmacol. (2024) 139:112766. doi: 10.1016/j.intimp.2024.112766, PMID: 39067403

[13] Jan Z El Assadi F Abd-Alrazaq A Jithesh PV . Artificial intelligence for the prediction and early diagnosis of pancreatic cancer: scoping review. J Med Internet Res. (2023) 25:e44248. doi: 10.2196/44248, PMID: 37000507 PMC10131763

[14] Wang P . Network biology: Recent advances and challenges. GPD 1. (2022) 1:101. doi: 10.36922/gpd.v1i2.101

[15] Guy A Morange PE James C . How I approach the treatment of thrombotic complications in patients with myeloproliferative neoplasms. Blood. (2025) 145:1769–79. doi: 10.1182/blood.2024025627, PMID: 39541574

[16] Marin Oyarzun CP Heller PG . Platelets as mediators of thromboinflammation in chronic myeloproliferative neoplasms. Front Immunol. (2019) 10:1373. doi: 10.3389/fimmu.2019.01373, PMID: 31258539 PMC6587101

[17] Ritchie ME Phipson B Wu D Hu Y Law CW Shi W . limma powers differential expression analyses for RNA-sequencing and microarray studies. Nucleic Acids Res. (2015) 43:e47. doi: 10.1093/nar/gkv007, PMID: 25605792 PMC4402510

[18] Kolde R Laur S Adler P Vilo J . Robust rank aggregation for gene list integration and meta-analysis. Bioinformatics. (2012) 28:573–80. doi: 10.1093/bioinformatics/btr709, PMID: 22247279 PMC3278763

[19] Leek JT Johnson WE Parker HS Jaffe AE Storey JD . The sva package for removing batch effects and other unwanted variation in high-throughput experiments. Bioinformatics. (2012) 28:882–3. doi: 10.1093/bioinformatics/bts034, PMID: 22257669 PMC3307112

[20] Yu G Wang LG Han Y He QY . clusterProfiler: an R package for comparing biological themes among gene clusters. OMICS. (2012) 16:284–7. doi: 10.1089/omi.2011.0118, PMID: 22455463 PMC3339379

[21] Khoury JD Solary E Abla O Akkari Y Alaggio R Apperley JF . The 5th edition of the world health organization classification of haematolymphoid tumours: myeloid and histiocytic/dendritic neoplasms. Leukemia. (2022) 36:1703–19. doi: 10.1038/s41375-022-01613-1, PMID: 35732831 PMC9252913

[22] Newman AM Liu CL Green MR Gentles AJ Feng W Xu Y . Robust enumeration of cell subsets from tissue expression profiles. Nat Methods. (2015) 12:453–7. doi: 10.1038/nmeth.3337, PMID: 25822800 PMC4739640

[23] Guglielmelli P Mora B Gesullo F Mannelli F Loscocco GG Signori L . Clinical impact of mutated JAK2 allele burden reduction in polycythemia vera and essential thrombocythemia. Am J Hematol. (2024) 99:1550–9. doi: 10.1002/ajh.27400, PMID: 38841874

[24] Yang B Qu D Zhao AL Li Y Meng RR Yu JX . Identification of differentially expressed genes in Budd−Chiari syndrome by RNA−sequencing. Mol Med Rep. (2017) 16:8011–8. doi: 10.3892/mmr.2017.7621, PMID: 28983615 PMC5779883

[25] Lu M Xia L Liu YC Hochman T Bizzari L Aruch D . Lipocalin produced by myelofibrosis cells affects the fate of both hematopoietic and marrow microenvironmental cells. Blood. (2015) 126:972–82. doi: 10.1182/blood-2014-12-618595, PMID: 26022238 PMC4543230

[26] Lu M Xia L Liu Y-C Hochman T Weinberg RS Goldberg JD . The effects of lipocalin (LCN2) on hematopoiesis in primary myelofibrosis. Blood. (2014) 124:1878. doi: 10.1182/blood.V124.21.1878.1878

[27] Xia Y Ge G Xiao H Wu M Wang T Gu C . REPIN1 regulates iron metabolism and osteoblast apoptosis in osteoporosis. Cell Death Dis. (2023) 14:631. doi: 10.1038/s41419-023-06160-w, PMID: 37749079 PMC10519990

[28] Saranya I Selvamurugan N . Regulation of TGF-β/BMP signaling during osteoblast development by non-coding RNAs: Potential therapeutic applications. Life Sci. (2024) 355:122969. doi: 10.1016/j.lfs.2024.122969, PMID: 39142506

[29] He M Ao X Yang Y Xu Y Liu T Ao L . Construction of self-driving anti-αFR CAR-engineered NK cells based on IFN-γ and TNF-α synergistically induced high expression of CXCL10. Neoplasia. (2024) 58:101065. doi: 10.1016/j.neo.2024.101065, PMID: 39366148 PMC11489333

[30] Mujal AM Owyong M Santosa EK Sauter JC Grassmann S Pedde AM . Splenic TNF-α signaling potentiates the innate-to-adaptive transition of antiviral NK cells. Immunity. (2025) 58:585–600.e6. doi: 10.1016/j.immuni.2025.02.012, PMID: 40023159 PMC12668197

[31] Riley CH Hansen M Brimnes MK Hasselbalch HC Bjerrum OW Straten PT . Expansion of circulating CD56bright natural killer cells in patients with JAK2-positive chronic myeloproliferative neoplasms during treatment with interferon-alpha. Eur J Haematol. (2015) 94:227–34. doi: 10.1111/ejh.12420, PMID: 25082025

[32] Lu ZQ Zhang C Zhao LJ Dong W Lv L Lu Y . Matrix metalloproteinase-8 regulates dendritic cell tolerance in late polymicrobial sepsis via the nuclear factor kappa-B p65/β-catenin pathway. Burns Trauma. (2024) 12:tkad025. doi: 10.1093/burnst/tkad025, PMID: 38425412 PMC10903637

[33] Vikman S Larsson A Thulin M Karlsson T . Increased levels of a subset of angiogenesis-related plasma proteins in essential thrombocythemia. Ups J Med Sci. (2023) 128. doi: 10.48101/ujms.v128.9194, PMID: 37051288 PMC10084492

[34] Flo TH Smith KD Sato S Rodriguez DJ Holmes MA Strong RK . Lipocalin 2 mediates an innate immune response to bacterial infection by sequestrating iron. Nature. (2004) 432:917–21. doi: 10.1038/nature03104, PMID: 15531878

[35] Balbín M Fueyo A Tester AM Pendás AM Pitiot AS Astudillo A . Loss of collagenase-2 confers increased skin tumor susceptibility to male mice. Nat Genet. (2003) 35:252–7. doi: 10.1038/ng1249, PMID: 14517555

