# Supplementary material for: Dysregulation of the immune microenvironment in essential thrombocythemia: the interplay of core genes and inflammation-related signaling pathways
Source: Front Immunol. 2026 Apr 29;17:1741533. doi: 10.3389/fimmu.2026.1741533 (PMC13167937; doi:10.3389/fimmu.2026.1741533)
Supplement: Supplementary file 1 [file Table1.docx]

Supplementary Material for “Dysregulation of the Immune Microenvironment in Essential Thrombocythemia: The Interplay of Core Genes and Inflammation-Related Signaling Pathways”

Supplementary Figures


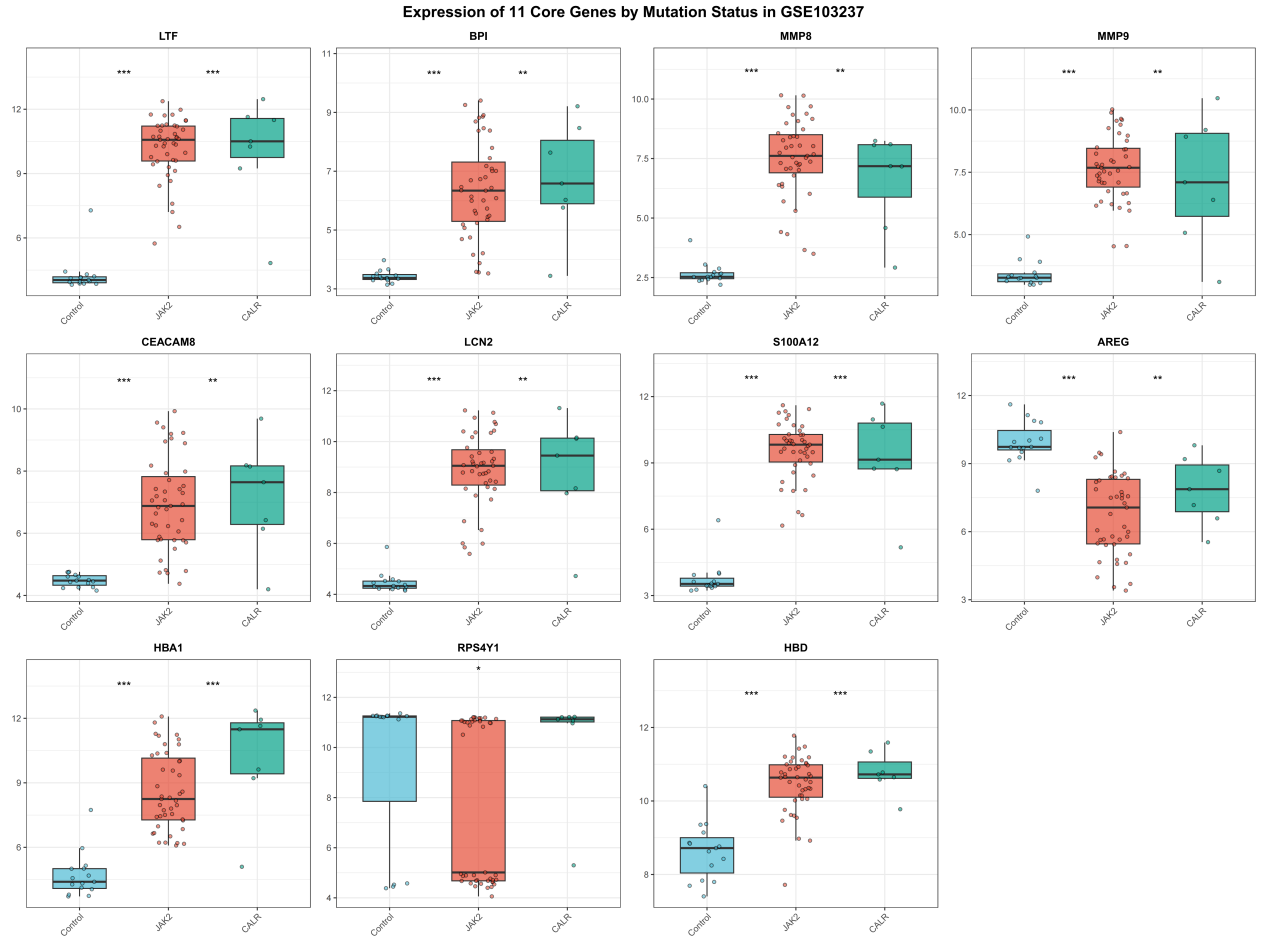


**Figure S1: Expression of 11 core genes by mutation status in GSE103237.**

# Supplementary Tables

**Table S1: Data sets used in this study.**

| GEO | Sample | Organization | Type | Platform |
| --- | --- | --- | --- | --- |
| GSE9827 | BM CD34+ cells from healthy subjects 4 samples ET JAK2V617F positive/negative 16 samples | Bone marrow (BM) CD34+ cells | Expression profiling by array | GPL96 |
| GSE2006 | Peripheral blood platelets from 6 ET patients and 5 healthy individuals | Peripheral Blood Platelets | Expression profiling by array | GPL96 |
| GSE103237 | 24 ET and 15 normal samples | Bone Marrow (BM) CD34+ cells | Expression profiling by array | GPL13667 |
| GSE54644 | 47 ET and 11 normal samples | Peripheral Blood Neutrophils | Expression profiling by array | GPL4685 |
| GSE136335 | 2 Et and 3 normal samples | Bone Marrow (BM) CD34+ Cells | Expression profiling by array | GPL17586 |
| GSE174060 | 6 ET and 6 healthy | Bone Marrow (BM) CD34+ Cells | Expression profiling by array | GPL17586 |
| GSE61629 | 8 ET and 21 healthy | Whole Blood | Expression profiling by array | GPL570 |

**Table S2: Sequences of primers used in this study.**

| Primer name | Primer sequences |
| --- | --- |
| GAPDH-F  GAPDH-R  LCN2-F  LCN2-R  LTF-F  LTF-R  BP1-F  BP1-R  MMP8-F  MMP8-R  MMP9-F  MMP9-R  CEACAM8-F  CEACAM8-R | 5'-AGCCACATCGCTCAGACAC-3'  5'-GCCCAATACGACCAAATCC-3'  5'-GAGTTACCCTGGATTAACGAG -3'  5'-AAGCGGATGAAGTTCTCCTTTA -3'  5'-GACCGAAAGACAGCCACGA-3'  5'-ACACAGGTTGGGGAACTGTC-3'  5'-CTCACCCAGACCCAGGTAAAG-3'  5'-ATCATCTGAGGCGAAGCCAG-3'  5'-GATTGCCCAGACATTTGTACTGTG-3'  5'-GTGGGACCAGGTTACATGATTTGA-3'  5'- ATTGCTTAAGCCCAGGAGTTCCAG-3'  5'-CTCAAAGACCGAGTCCAGCTTG-3'  5'-CCTATAATTCGACGGTTGGCAT-3'  5'-TGCTCAGCTCACTATTGAAGC-3' |

**Table S3: Differential genes analyzed by RRA.**

| Name | Pvalue | FDR | logFC |
| --- | --- | --- | --- |
| HBD | 7.10E-16 | 2.75E-11 | 1.429859 |
| RPS4Y1 | 3.19E-15 | 1.23E-10 | -1.56556 |
| AREG | 6.81E-14 | 1.32E-09 | -2.23541 |
| HBG1 | 7.94E-10 | 7.69E-06 | 1.389715 |
| EIF1AY | 1.05E-09 | 1.02E-05 | -1.01689 |
| DDX3Y | 2.60E-09 | 1.68E-05 | -1.08527 |
| XIST | 4.05E-09 | 3.14E-05 | 1.16937 |
| LTF | 4.90E-08 | 0.000145 | 1.223754 |
| BLVRB | 6.39E-08 | 0.000165 | 1.002843 |
| FGL2 | 1.65E-07 | 0.000329 | 1.078677 |
| HBA1 | 2.23E-07 | 0.000351 | 1.128047 |
| XK | 5.13E-07 | 0.000584 | 1.023298 |
| LCN2 | 6.25E-07 | 0.000642 | 1.23113 |
| MMP8 | 8.55E-07 | 0.000719 | 1.045752 |
| CYBB | 9.07E-07 | 0.000745 | 1.08092 |
| BPI | 1.07E-06 | 0.000825 | 1.003036 |
| CD163 | 2.95E-06 | 0.001628 | 1.154309 |
| CD68 | 4.78E-06 | 0.002089 | 1.05998 |
| TIMD4 | 4.80E-06 | 0.002089 | 1.391782 |
| CEACAM8 | 5.25E-06 | 0.002186 | 1.073499 |
| SPIC | 7.93E-06 | 0.002968 | 1.144474 |
| PROS1 | 8.07E-06 | 0.002968 | 1.066674 |
| MS4A6A | 1.25E-05 | 0.003826 | 1.090221 |
| LGMN | 1.25E-05 | 0.003826 | 1.458269 |
| MMP9 | 1.61E-05 | 0.004539 | 1.014358 |
| S100A12 | 1.92E-05 | 0.005105 | 1.403107 |
| FCGR2A | 2.27E-05 | 0.005491 | 1.008004 |
| VCAM1 | 2.47E-05 | 0.00572 | 1.136241 |
| HPSE | 4.29E-05 | 0.008221 | 1.0606 |
| A2M | 7.67E-05 | 0.011463 | 1.042059 |

**Table S4: Analysis of Differences in Immune Cell Proportion**

| **cells** | **P** | **FDR** |
| --- | --- | --- |
| B cells naive | 0.00013 | 0.00286 |
| T cells CD4 memory resting | 0.00028 | 0.00308 |
| NK cells activated | 0.002 | 0.01467 |
| Macrophages M0 | 0.005 | 0.0275 |
| T cells CD4 memory activated | 0.007 | 0.0308 |
| NK cells gamma delta | 0.021 | 0.077 |
| Eosinophils | 0.023 | 0.07229 |
| Plasma cells | 0.028 | 0.077 |
| Mast cells activated | 0.028 | 0.06844 |
| B cells memory | 0.035 | 0.077 |
| Macrophages M1 | 0.059 | 0.118 |
| T cells CD4 naive | 0.062 | 0.11367 |
| NK cells resting | 0.094 | 0.15908 |
| Macrophages M2 | 0.111 | 0.17443 |
| T cells regulatory (Tregs) | 0.131 | 0.19213 |
| T cells CD8 | 0.162 | 0.22275 |
| Neutrophils | 0.177 | 0.22906 |
| Monocytes | 0.25 | 0.30556 |
| Dendritic cells resting | 0.376 | 0.43537 |
| Mast cells resting | 0.45 | 0.495 |
| T cells follicular helper | 0.716 | 0.74952 |
| Dendritic cells activated | 0.896 | 0.896 |

**Table S5: Correlation analysis between genes and cells**

| **Gene** | **cells** | **P** | **FDR** | **R** |
| --- | --- | --- | --- | --- |
| MMP9 | Macrophages M0 | 1.7e-13 | 1.36e-12 | 0.64 |
| MMP8 | Macrophages M0 | 1.1e-08 | 4.40e-08 | 0.52 |
| CEACAM8 | Macrophages M0 | 7.8e-08 | 2.08e-07 | 0.49 |
| LCN2 | Macrophages M0 | 7.1e-06 | 1.42e-05 | 0.49 |
| S100A12 | Eosinophils | 3.5e-07 | 5.60e-07 | -0.47 |
| LTF | NK cells activated | 1.2e-06 | 1.60e-06 | -0.45 |
| MMP8 | NK cells activated | 2.6e-06 | 2.97e-06 | -0.44 |
| MMP9 | T cells CD4 memory resting | 3.2e-06 | 3.20e-06 | -0.43 |

**Table S6: Patient Information**

| **Patient ID** | **Age** | **Sex** | **JAK2 mutation** | **CALR mutation** | **MPL mutation** | **Treatment** |
| --- | --- | --- | --- | --- | --- | --- |
| ET patients (n=20) | | | | | | |
| ET-01 | 58 | Male | Positive | Negative | Negative | None |
| ET-02 | 62 | Female | Negative | Positive | Negative | Hydroxyurea |
| ET-03 | 45 | Male | Positive | Negative | Negative | None |
| ET-04 | 51 | Female | Positive | Negative | Negative | Aspirin |
| ET-05 | 49 | Male | Negative | Positive | Negative | None |
| ET-06 | 63 | Female | Positive | Negative | Negative | Hydroxyurea |
| ET-07 | 55 | Female | Negative | Negative | Negative | Interferon-α |
| ET-08 | 60 | Female | Positive | Negative | Negative | None |
| ET-09 | 47 | Male | Negative | Positive | Negative | Aspirin |
| ET-10 | 52 | Male | Positive | Negative | Negative | Hydroxyurea |
| ET-11 | 68 | Female | Positive | Negative | Negative | None |
| ET-12 | 41 | Female | Negative | Positive | Negative | Hydroxyurea |
| ET-13 | 57 | Female | Positive | Negative | Negative | Aspirin |
| ET-14 | 64 | Female | Negative | Negative | Positive | None |
| ET-15 | 50 | Male | Positive | Negative | Negative | Hydroxyurea |
| ET-16 | 59 | Female | Negative | Positive | Negative | Interferon-α |
| ET-17 | 46 | Female | Positive | Negative | Negative | None |
| ET-18 | 53 | Male | Positive | Negative | Negative | Aspirin |
| ET-19 | 61 | Male | Negative | Negative | Negative | Hydroxyurea |
| ET-20 | 48 | Female | Positive | Negative | Negative | None |
| Healthy controls (n=20) | | | | | | |
| Control-01 | 55 | Male | - | - | - | - |
| Control-02 | 48 | Female | - | - | - | - |
| Control-03 | 42 | Male | - | - | - | - |
| Control-04 | 39 | Female | - | - | - | - |
| Control-05 | 61 | Male | - | - | - | - |
| Control-06 | 44 | Female | - | - | - | - |
| Control-07 | 53 | Male | - | - | - | - |
| Control-08 | 49 | Female | - | - | - | - |
| Control-09 | 56 | Male | - | - | - | - |
| Control-10 | 38 | Female | - | - | - | - |
| Control-11 | 60 | Male | - | - | - | - |
| Control-12 | 47 | Female | - | - | - | - |
| Control-13 | 52 | Male | - | - | - | - |
| Control-14 | 43 | Female | - | - | - | - |
| Control-15 | 59 | Male | - | - | - | - |
| Control-16 | 46 | Female | - | - | - | - |
| Control-17 | 54 | Male | - | - | - | - |
| Control-18 | 41 | Female | - | - | - | - |
| Control-19 | 57 | Male | - | - | - | - |
| Control-20 | 50 | Female | - | - | - | - |
